# Quantitative approach for cardiac risk assessment and interpretation in tuberculosis drug development

**DOI:** 10.1007/s10928-018-9580-2

**Published:** 2018-03-08

**Authors:** Sebastian Polak, Klaus Romero, Alexander Berg, Nikunjkumar Patel, Masoud Jamei, David Hermann, Debra Hanna

**Affiliations:** 1 Certara-Simcyp, Level 2-Acero, 1 Concourse Way, Sheffield, S1 2BJ UK; 2 Unit of Pharmacoepidemiology and Pharmacoeconomics, Department of Social Pharmacy, Faculty of Pharmacy, Jagiellonian University Medical College, Medyczna 9, Kraków, 30-217 Poland; 3 Critical Path Institute, 1730 E. River Road, Tucson, AZ 85705 USA; 4 Certara USA, Inc, Princeton, NJ USA

**Keywords:** Torsade de pointes, Drugs, Tuberculosis, Cardiac risk, Cardiac safety simulator, Simcyp, Model, Simulation

## Abstract

Cardiotoxicity is among the top drug safety concerns, and is of specific interest in tuberculosis, where this is a known or potential adverse event of current and emerging treatment regimens. As there is a need for a tool, beyond the QT interval, to quantify cardiotoxicity early in drug development, an empirical decision tree based classifier was developed to predict the risk of Torsades de pointes (TdP). The cardiac risk algorithm was developed using pseudo-electrocardiogram (ECG) outputs derived from cardiac myocyte electromechanical model simulations of increasing concentrations of 96 reference compounds which represented a range of clinical TdP risk. The algorithm correctly classified 89% of reference compounds with moderate sensitivity and high specificity (71 and 96%, respectively) as well as 10 out of 12 external validation compounds and the anti-TB drugs moxifloxacin and bedaquiline. The cardiac risk algorithm is suitable to help inform early drug development decisions in TB and will evolve with the addition of emerging data.

**Electronic supplementary material:**

The online version of this article (10.1007/s10928-018-9580-2) contains supplementary material, which is available to authorized users.

## Introduction

### The need for new drugs against tuberculosis

Tuberculosis (TB) is a condition that affects one-quarter of the world’s population, causing 1.5 million TB-related deaths per year and remains the leading world-wide cause of death due to an infectious disease [1]. Due to the protracted duration of anti-TB therapy, combination drug regimens are needed that enable a shorter treatment duration while maintaining an acceptable safety profile. This necessitates both a robust drug development pipeline, as well as an improved drug development process. Drug development tools that provide improved predictions of efficacy and safety for combination regimens are expected to enhance the efficiency of regimen development process by informing key decisions.

Since its inception in 2010, the critical path to TB drug regimen (CPTR) initiative, a global public–private-partnership, has keenly focused on accelerating the development of an entirely novel, shorter duration therapy for TB [2]. A core aspect of CPTR’s strategy is the development, validation and refinement of a suite of pre-clinical, translational methodologies and quantitative drug development platforms. These efforts are focused on optimizing the translation of novel TB drugs by leveraging integrated and standardized data to develop first-in-class translational methodologies, including the cardiac risk algorithm presented in this manuscript.

### Efficient drug safety assessment during development

Characterization of drug safety, equally with drug efficacy, is critical during the drug development process. Prior to human testing, non-clinical safety pharmacology and toxicology studies are conducted to predict compound risks in humans, focusing on the risk of rare lethal events [3]. Despite extensive non-clinical testing, toxicological and safety-related issues are the most common reasons for drug candidate attrition up to Phase 2 [4]. Cardiotoxicity and hepatotoxicity are among the top safety concerns, yet comprehensive, detailed data on these toxicities is still sparse in the public space [5, 6]. While the latter is a common toxicity of anti-TB therapy, especially for first-line drugs like isoniazid and rifampicin [7], electrophysiological disruption of cardiac activity represents another main concern for drug development in general, and for anti-TB drug development specifically.

Torsade de pointes (TdP) is a syndrome of polymorphic ventricular arrhythmia occurring in the setting of marked prolongation of the QT interval as assessed by electrocardiogram (ECG). In TdP the QT interval is prolonged in the heartbeats before the sudden onset of rapid and disorganized contractions of the heart. Patients with TdP experience dizziness or loss of consciousness if the arrhythmia is brief. If sustained, TdP can be lethal. Certain clinical conditions which prolong the QT interval, such as congenital long QT syndrome or the administration of drugs that block cardiac potassium channels, are often associated with TdP, although it may not manifest at the time QT prolongation is observed. Given this association, the occurrence of post-dose QT prolongation is a commonly used biomarker to identify drugs that could result in iatrogenic TdP.

There is growing awareness that QT or heart-rate corrected QT interval (QTc) prolongation is a limited or incomplete biomarker of TdP risk. In some cases, QT prolongation secondary to drug administration is not an accurate indicator of TdP risk and therefore may not be informative in the context of drug development [8]. Sponsors, academicians, and regulators agree that a more comprehensive characterization of risk, based on complete cardiac ion channel profiles and thorough quantification of electrophysiological effects, may provide better concordance between ECG signals and TdP risk [9, 10].

To help address this challenge, multiple classification models were proposed recently using various, often heterogenic sets of data representing a range of input variables [11–13]. A more comprehensive analysis of clinical data to enhance the current ECG assessment was recently proposed by Johannesen and colleagues [14]. They hypothesized that the effects of multichannel drug block can be described by the thorough analysis of early repolarization (J–T_peak_; the duration between the J point and the peak of the T wave) and late repolarization (T_peak_–T_end_; the duration between the peak and the end of the T wave). In a clinical study led by Johannesen, four drugs were given to 22 healthy volunteers, dofetilide (a pure human ether-a-go–go-related gene (hERG) potassium channel blocker), quinidine, ranolazine, and verapamil (as drugs that block hERG and either calcium or late sodium currents). The results showed that a more thorough ECG-based characterization of multichannel drug effects on human cardiac repolarization may improve the assessment of drug-related cardiac electrophysiology disruption.

### Rationale of the project and aim of the study

Given the reliance on drug combinations for effective TB treatment and the large proportion of standard anti-TB agents which are known or suspected to be torsadogenic, a robust cardiac safety testing platform should be utilized in the development of new and novel anti-TB agents. This study details the development of component of a quantitative cardiac safety testing platform, which was conducted according to four specific aims:

1. Collate available clinical and non-clinical data pertaining to adverse cardiovascular effects and associated clinical outcomes of anti-TB drugs (and their metabolites) when used individually and in combination (e.g., cardiac ion channel activity profiles, repolarization reserve effects—QT changes, T-wave morphology changes, autonomic tone effects, TdP, sudden death, hospitalization, etc.);
2. Develop an empirical TdP risk assessment model for single chemical moieties;
3. Incorporate subject-level electrophysiological data with complete ion channel activity profiles of QT-prolonging drugs into a quantitative tool for use in the development of comprehensive algorithms characterizing cardiac arrhythmia risk profiles for anti-TB drug combinations;
4. Inform experimental designs (in vitro, preclinical, and clinical), data analytics, signal interpretation, and go/no-go decisions related to cardiac arrhythmia risk in the context of anti-TB drug combination development.

The purpose of the work presented herein is to provide drug developers with a tool for use early in anti-TB drug development, to better quantify the risk of TdP utilizing in vitro ion current inhibition results in combination with human PK. Specifically, this report summarizes Aims 1 and 2 outlined above; namely, the development of an in silico model, the cardiac risk algorithm, an empirical classifier for single chemical moieties intended to support quantitative TdP risk assessment, based on the cardiac electrophysiology of human left ventricular cardiomyocytes along with a quantitative assessment of TdP risk.

## Methods

The centerpiece of the Cardiac Risk Algorithm is the in silico model of the ventricular myocyte as implemented in the Simcyp cardiac safety simulator (CSS 2.0, Simcyp, Certara). The CSS includes two biophysically-detailed myocyte models (BDMM) describing electrophysiology of the human left ventricular cardiomyocytes which are considered to represent state-of-the-art models based on the Hodgkin-Huxley action potential formalism [15, 16]. The model by ten Tusscher (tT04) represents the default [17]. Additionally, the updated ten Tusscher (tT06) and O’Hara-Rudy models (ORD) [18, 19] were also implemented. Both models were developed from data derived predominantly (tT) or exclusively (ORD) from experiments using human myocytes. The forward Euler method is employed to integrate model equations.

CSS operates on two levels, simulating either single cell electrophysiology and outputting action potential and its derivatives (APD50, APD90) or simulating a one-dimensional string of cells, where the output is a pseudoECG (viz. an in silico ECG) and its derivatives (e.g., QRS, QT). In the latter case, as a one-dimensional fiber of cardiomyocytes is heterogeneous in character, to mimic the human heart, CSS has a left ventricular wall thickness comprised by default of 50% endocardial, 30% midmyocardial and 20% epicardial cells. All other physiological parameters describe virtual individuals, using known cardiomyocyte morphometric parameters (volume, area, electric capacitance). Plasma ion concentration (K^+^, Na^+^, Ca^2+^) and heart rate are specific for healthy individuals [25–27]. CSS accounts for intra-individual circadian variability in physiological parameters (e.g., heart rate, plasma ion concentrations) [28]. Every element of the CSS system, including physiological parameters and their variability, was obtained from the scientific literature as reported in a peer reviewed publication or white paper [20]. CSS has itself been validated in a growing number of publications [15, 21, 22]. The CSS predictive performance was based on the comparison of the simulated and clinically observed endpoints (e.g., action potential duration [APD], QT, QRS) [23].

Data inputs required for the CSS are as follows:

1. Nonclinical ionic channel inhibitory effects as measured by patch clamp assay (i.e., IC_50_ values for rapidly activating delayed rectifier potassium current IKr (encoded by hERG gene), delayed rectifier potassium current IKs (encoded by KvLQT1/mink gene), peak sodium current INa (encoded by Nav1.5 gene), L-type calcium current ICa (encoded by Cav1.2 gene) and other currents if available);
2. Drug concentrations of parent and/or active metabolites and/or concomitantly given drugs as obtained from clinical studies or simulated using the Simcyp physiologically based pharmacokinetic (PBPK) model; and
3. Subject specific covariates affecting cardiac myocyte APD or drug exposure (i.e., volume of cardiac myocytes, sarcoplastic reticulum volume, cardiomyocytes electric capacitance, plasma ions concentration, heart rate) [25–27].

Drug effects on four main ion channels were simulated with a simple pore block model. All input data for the drugs provided in this manuscript are available in the Supplementary files.

Available outputs from the Simcyp CSS module include:

1. Single cell action potential and its derivatives (APD50, APD90—the time needed for 50 and 90% of the cell repolarization)
2. PseudoECG signal and its derivatives (QRS, QT/QT_c_, J-T_peak_, and T_peak_ − T_end_)
3. Electro-mechanical window (time gap between the end of electric and mechanical systole)

These outputs were utilized to develop models of proarrhythmic potency (cardiac risk algorithms) for single drugs or drug combinations. Multiple machine learning algorithms implemented in the Waikato environment for knowledge analysis (WEKA) were tested during model development, including decision trees, random forests, and support vector machines [24]. In all scenarios, the same procedure was applied, namely the data set was randomly divided to the learning set (90% of cases) and testing set (10% of cases) in a tenfold cross validation procedure, and eventually a validation set of 12 compounds was evaluated using the best obtained model. The cardiac risk algorithm output is continuous in the range < 0;1 > and is interpreted as the probability of a compound lacking an association with TdP. A threshold of 0.5 was chosen as a default value; therefore all results ≥ 0.5 were classified as negative and those < 0.5 as positive (viz., TdP(−) and TdP(+), respectively).

**Fig. 1:**
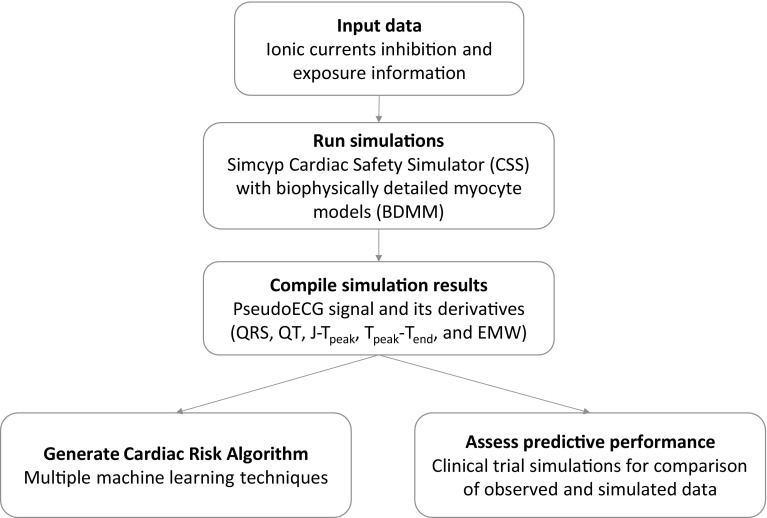
Model building schema

The analysis plan consisted of the following steps (Fig. 1):

1. Stage 1: Development of cardiac risk algorithm
2. Stage 2: Evaluation of the cardiac risk algorithm on validation dataset
3. Stage 3: Application of the proposed cardiac risk algorithm to the chosen anti-TB drugs (moxifloxacin, bedaquiline)

Data describing the in vitro measured inhibition of all ionic channels were collected from the available literature for 12 validation drugs and bedaquiline. This included information about multiple ionic current inhibition measured via the patch clamp technique. All collected data are provided in the supplementary materials (Supplementary Table 1), including estimated IC_50_ values and detailed descriptions of the measurement settings which differ for the various ionic currents. The IC_50_ values applied during the simulation study are marked in green and were selected based on the assumption that the in vitro study should mimic the in vivo human physiology as closely as possible. The main parameters included: temperature, ion concentrations, and electric protocol (i.e., holding potential [mV], depolarization potential [mV], repolarization potential [mV], and measurement potential [mV]).

Simulations were performed in the CSS at the pseudoECG level in one female virtual individual, representative of the Caucasian population (viz. “PopRep”), with 10 concentrations simulated for each drug (0, 0.0001, 0.001, 0.01, 0.1, 1, 10, 30, 100, and 500 µM). The randomly derived physiological parameters characterizing the virtual individual are listed below in Table 1 [25–29].

**Table 1:** PopRep virtual individual characteristics

Parameter	Value	Unit
Age	34	Years
Plasma potassium concentration (K^+^)	5.49	mM
Plasma sodium concentration (Na^+^)	137.59	mM
Plasma calcium concentration (Ca^2+^)	2.25	mM
RR	808	ms
Cardiomyocyte volume	8183	µm^3^
Cardiomyocyte area	2274	µm^2^
Electric capacitance	60.42	pF
Sarcoplasmic reticulum volume	628.1	µm^3^
String length (heart wall thickness)	10.8	mm

Additional endpoints for further analysis were simulated using the CSS, including:

- QT_c_ and/or QT_c_ prolongation (difference between baseline and drug modified QT_c_)
- QRS and/or QRS prolongation (difference between baseline and drug modified QRS)
- Index of cardiac electrophysiological balance (iCEB; = QT/QRS) [30]
- Electromechanical window (EMW) [31]

**Fig. 2:**
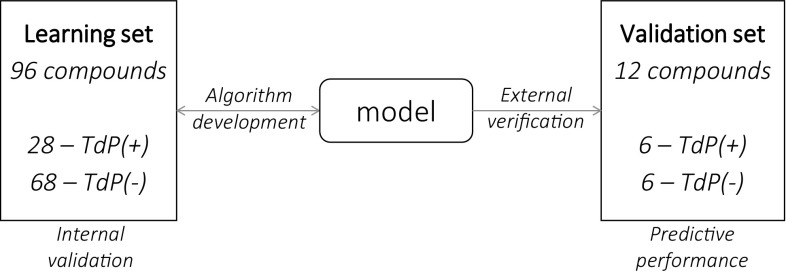
Drugs classification and application in algorithm development

For cardiac risk algorithm development, the learning set consisted of 96 compounds (presented in Supplementary material) and 12 compounds (Table 2) were used to assess model predictive performance. All compounds were classified as TdP(+) or TdP(−) based on the CredibleMeds^®^ QTdrugs classification as of November 2014 (Fig. 2, Supplementary Table 1) [32, 33].

**Table 2:** Validation drugs classification

Drug name	CredibleMeds classification for TdP Risk	Binary TdP classification
Fexofenadine	Not mentioned	Negative
Propafenone	Not mentioned	Negative
Verapamil	Not mentioned	Negative
Hydrodolasetron	Possible risk of TdP (known association with QT prolongation, unknown association with TdP)	Negative
Ranolazine	Possible risk of TdP (known association with QT prolongation, unknown association with TdP)	Negative
Vardenafil	Possible risk of TdP (known association with QT prolongation, unknown association with TdP)	Negative
Amiodarone	Known risk of TdP (known association with TdP)	Positive
Citalopram	Known risk of TdP (known association with TdP)	Positive
Clarithromycin	Known risk of TdP (known association with TdP)	Positive
Dofetilide	Known risk of TdP (known association with TdP)	Positive
Moxifloxacin	Known risk of TdP (known association with TdP)	Positive
Quinidine	Known risk of TdP (known association with TdP)	Positive

According to CredibleMeds^®^, drugs are classified as:

1. Known Risk of TdP—these drugs prolong the QT interval AND are clearly associated with a known risk of TdP, even when taken as recommended (here classified as TdP(+) or positive).
2. Possible Risk of TdP—these drugs can cause QT prolongation BUT currently lack evidence for a risk of TdP when taken as recommended (here classified as TdP(−) or negative).
3. Conditional Risk of TdP—these drugs are associated with TdP BUT only under certain circumstances of their use (i.e., excessive dose, in patients with conditions such as hypokalemia, or when taken with interacting drugs) OR by creating conditions that facilitate or induce TdP i.e., by inhibiting metabolism of a QT-prolonging drug or by causing an electrolyte disturbance that induces TdP (here classified as TdP(−) or negative).

Drugs which were not mentioned in the CredibleMeds^®^ listing were classified as TdP(−) or negative.

Among 96 compounds included in the learning dataset, 28 were classified as TdP(+) and 68 as TdP(−), whereas in the validation dataset the TdP(+) and TdP(−) compounds were balanced with 6 in each group (Fig. 2).

TB drugs of interest include moxifloxacin (Known Risk of TdP − TdP(+)) as an element of the validation set and bedaquiline (Possible Risk of TdP − TdP(−)) as a new compound.

## Results

**Fig. 3:**
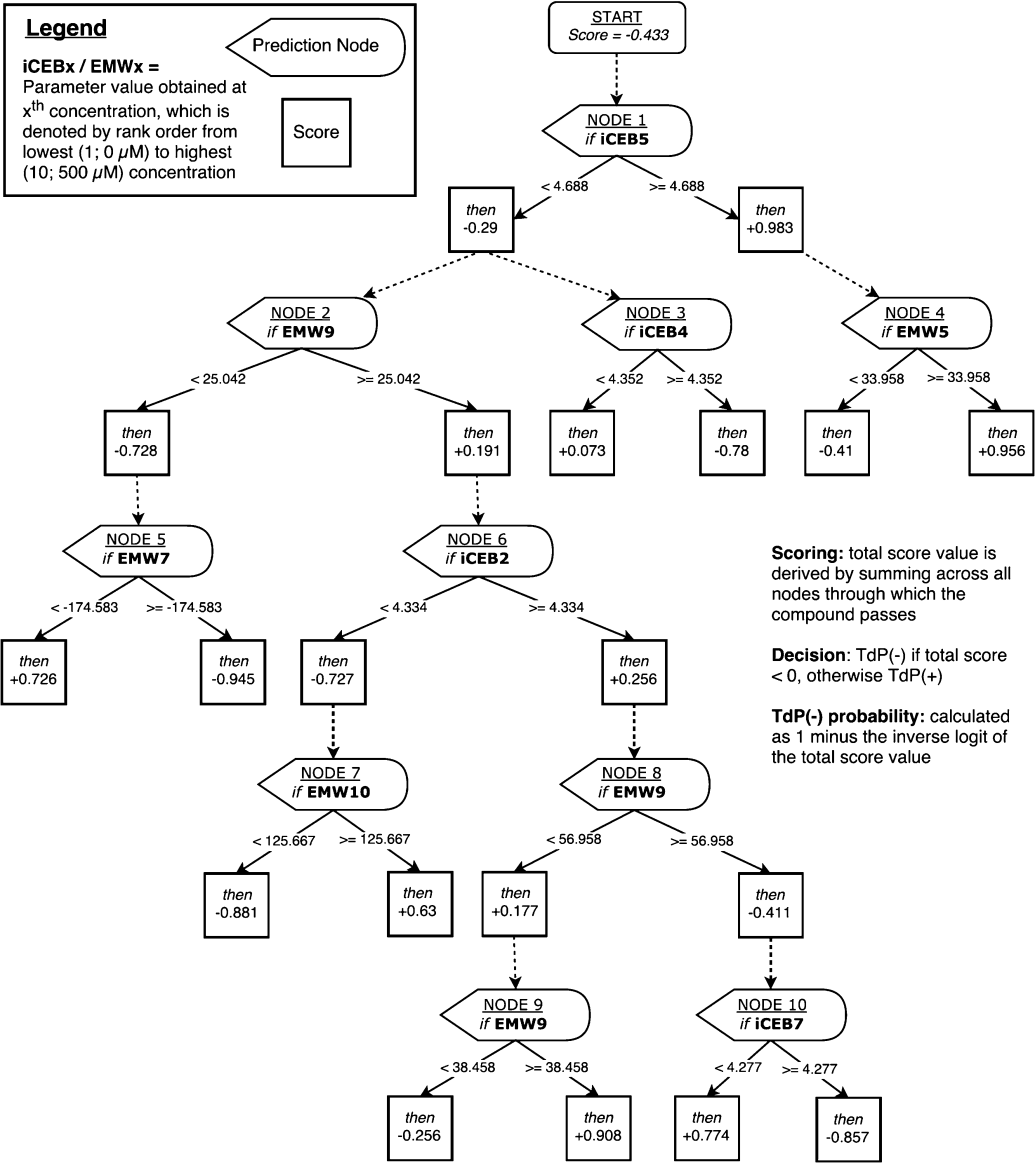
Drugs classification (from the WEKA classifier)

The best model (Fig. 3) was developed with the use of an alternating decision tree (ADTree) with input variables including EM window and iCEB calculated across 10 concentrations/compound (encoded consecutively as EMW1-10 and iCEB1-10) [34].

**Fig. 4:**
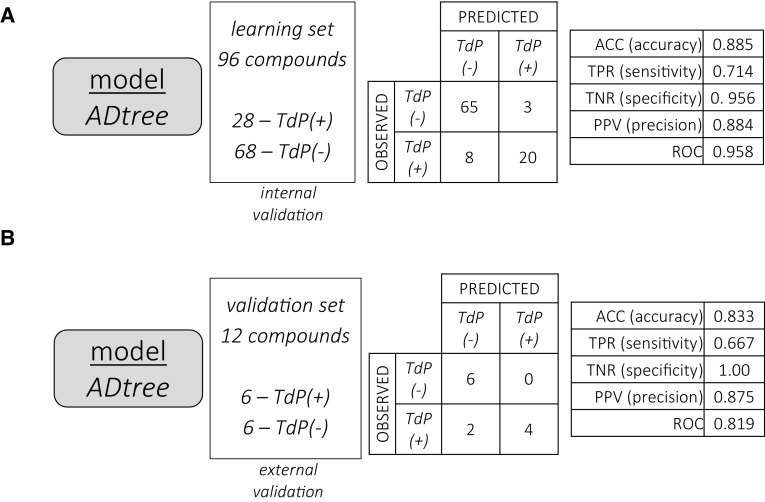
Results of the ADtree model—internal and external validation

**Fig. 5:**
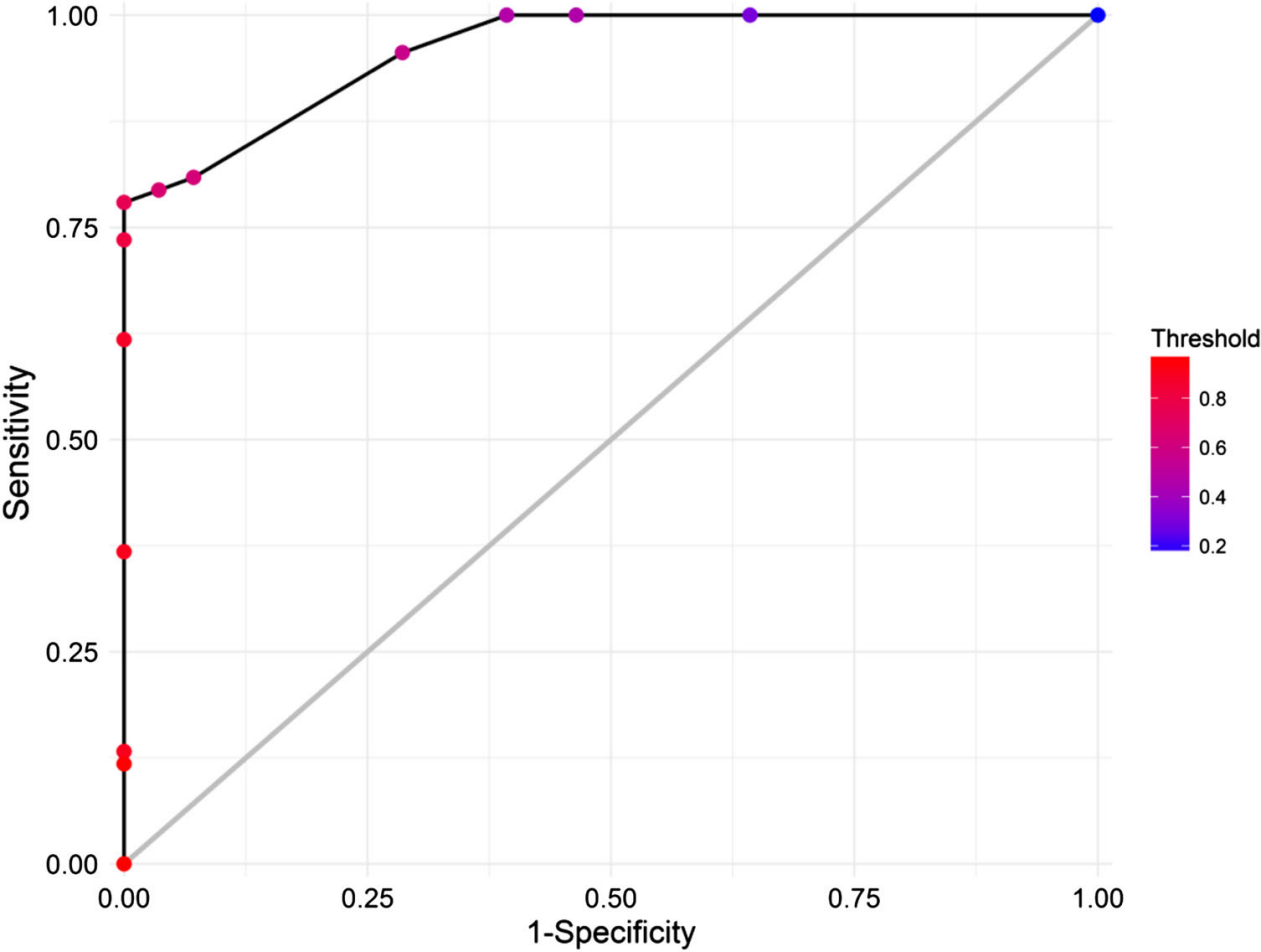
Receiver-operating curve for learning set at varying binary classification threshold values

The model correctly classified the TdP propensity of 85 out of 96 learning set compounds as assessed by internal validation procedures (89% accuracy), and 10 out of 12 compounds in the external validation set (83% accuracy). Results are presented in Fig. 4, Panels A and B respectively, whereas the receiver-operator curve (ROC) for the learning set is shown in Fig. 5.

The incorrectly classified drugs from the learning dataset are presented in Table 3. Of the compounds from the validation set, the incorrectly classified drugs included citalopram and quinidine which were both classified as TdP(−) or non-proarrhythmic. Both anti-TB drugs of interest were correctly classified according to their CredibleMeds^®^-based assessment, with moxifloxacin classified as TdP(+) and bedaquiline classified as TdP(−) with a high probability at 0.911.

**Table 3:** ADTree model incorrectly classified drugs from the learning dataset

Drug	Observed	Predicted	TdP(+) probability	TdP(−) probability
Gatifloxacin	TdP(−)	TdP(+)	0.535	0.465
Sertindole	TdP(−)	TdP(+)	0.535	0.465
Tolterodine	TdP(−)	TdP(+)	0.535	0.465
Chlorpromazine	TdP(+)	TdP(−)	0.342	0.658
Sotalol	TdP(+)	TdP(−)	0.364	0.636
Chloroquine	TdP(+)	TdP(−)	0.429	0.571
Disopyramide	TdP(+)	TdP(−)	0.429	0.571
Mesoridazine	TdP(+)	TdP(−)	0.429	0.571
Pentamidine	TdP(+)	TdP(−)	0.429	0.571
Probucol	TdP(+)	TdP(−)	0.429	0.571
Sparfloxacin	TdP(+)	TdP(−)	0.429	0.571

## Discussion

The cardiac risk algorithm describes the first step in the development of a comprehensive cardiac safety assessment platform to inform drug development decisions, in terms of the potential torsadogenic risk of candidate anti-TB drugs. This platform applies preclinical knowledge for different types of compounds, in terms of their ion channel activity and exposure–response relationships for electrophysiological activity, to generate a binary classification as to the potential risk of drug-induced TdP. As with any quantitative drug development platform, the Cardiac Risk Algorithm is intended to continuously evolve with additional data.

While the algorithm itself is empirical, it was derived from mechanistic information about multiple potentially affected channels and subsequent disruption of the electrical and mechanical cardiac activity. To capture the mechanistic model components, it was necessary to include all available information on the concentration-dependent inhibition of relevant cardiac ionic currents, which has potential limitations given the heterogeneity of the input data for the relevant parameters. Hence, strict criteria for utilization of the IC_50_ values were proposed a priori to reduce its influence on the simulation results and therefore the model quality. The applied selection criteria were that the in vitro study should mimic the in vivo human physiology as closely as possible, and that the lowest IC_50_ value should be applied. By applying these criteria, the mechanistic model inputs are believed to have been selected consistently, and albeit somewhat conservatively given the selection of the lowest reported IC_50_ value.

Although this platform is ultimately intended to support the assessment of novel and repurposed compounds for the treatment of TB, a systematic approach was used to merge in silico electromechanical simulations with observed clinical TdP risk for a range of clinically-relevant drugs across multiple therapeutic areas. This approach thereby incorporates drugs representing a range of structural classes with a broad spectrum of on- and off-target pharmacological effects. Of the clinically and structurally diverse compounds included in the 96 compound learning set, a total of 11 compounds were misclassified, with 3 incorrectly predicted as TdP(+) and 8 incorrectly predicted as TdP(−). In the former case, the three misclassified drugs, gatifloxacin, sertindole, and tolterodine belong to different therapeutic and structural classes and exhibited predicted probabilities of 0.47, barely below the minimum TdP(−) threshold value of 0.5. It is noted that since the time of algorithm development, gatifloxacin (a fluoroquinolone investigated for the treatment of TB) has been re-classified by CredibleMeds^®^ as a TdP(+) drug, supporting its corresponding classification in the present work [35]. Moreover, the CredibleMeds^®^ classification of sertindole and tolterodine as “Possible risk of TdP” is not necessarily inconsistent with mid-range probabilities and it is possible that, as in the case of gatifloxacin, additional clinical data may result in reclassification of these agents. For example, a sizeable proportion of sertindole-associated TdP cases have been reported in the context of overdoses; whereas tolterodine, as a CYP2D6 and CYP3A4 substrate, is a potential candidate for drug–drug-interactions that could contribute to TdP risk, although it remains to be seen if this drug has a potential to cause TdP when exposure magnitudes do not surpass therapeutic levels [36, 37]. Using only QT prolongation as the metric of TdP risk, 48 of the 96 test compounds would have been classified as having a risk of TdP whereas only 28 were truly associated with TdP. The cardiac risk algorithm (CRA) performed substantially better and appears to be a significant advance in assessing cardiac safety.

The 8 drugs in the learning set that were incorrectly classified as TdP(−) were structurally distinct, represented a range of therapeutic classes, and also exhibited probabilities of being TdP(−) close to the threshold value (range 0.57–0.66). Such “borderline” findings are not accounted for when converting the Cardiac Risk Algorithm probabilities to a binary TdP(+)/(−) classifier, and suggest that a different threshold value and/or a more discrete classification may be required.

The performance of the cardiac risk algorithm for the validation set compounds classified verapamil, fexofenadine, hydrodolasetron, vardenafil and ranolazine as having a high probability (> 0.8) of being TdP(−). The classification is in concordance with the available literature reports for verapamil, fexofenadine, and vardenafil, and it is consistent with ranolazine’s current CredibleMeds^®^ classification as being only a conditional risk drug [38–40]. Propafenone (which is not included in the CredibleMeds^®^ database and thus was treated as TdP(−) in this analysis) was also classified as lacking an association with TdP, but with mid-range probability [0.65 for TdP(−), 0.35 for TdP(+)]. This mid-range probability may be related to the pharmacological properties of this agent, as it is classified as a Class 1 antiarrhythmic and is known to be a moderate QT prolonging agent. Interestingly, another Class 1 antiarrhythmic included in the learning set, disopyramide, exhibited a similar probability of TdP(−) but was considered misclassified as it has a known risk of TdP according to CredibleMeds^®^. Thus, it is possible that propafenone may best be considered as having a “probable risk of TdP”, consistent with its mid-range probability per the Cardiac Risk Algorithm.

The two anti-TB drugs of interest, moxifloxacin and bedaquiline, were correctly classified as TdP(+) and TdP(−), respectively. Notably, moxifloxacin, a well-known QT prolonger, exhibited a mid-range TdP(−) probability of 0.46. While CredibleMeds^®^ classifies moxifloxacin as TdP(+), this drug is considered to be relatively safe as evidenced by its extensive use as a positive control for thorough QT (TQT) trials [40, 41]. Therefore, the probability predicted by the Cardiac Risk Algorithm seems appropriate, and suggests that moxifloxacin could be classified as a middle risk compound. Bedaquiline was classified as TdP(−), consistent with the lack of TdP reported in bedaquiline clinical trials [42].

Although the percentage of drugs misclassified by the proposed Cardiac Risk Algorithm is small (> 83% accuracy), it was considered that an alternative threshold value for the binary classification may improve the classification. However, the ROC analysis demonstrated that the threshold TdP(−) probability value of 0.5 used in this study was near optimal for the learning and testing sets of data. For example, while 100% of true TdP(−) compounds in the learning set can be correctly classified by decreasing the threshold probability slightly to 0.47, there is no change in the total number of incorrectly classified drugs as 11 known TdP(+) drugs are classified as TdP(−) with this threshold. Further, this alternative threshold does not modify the validation test results.

It may be logical to instead consider whether the results suggest that there is a need for at least three risk categories. From the observations, while it seems obvious that the intermediate risk category would allow not only better classification but also better risk assessment, the exact threshold values for trinary classification are not clear. More importantly, classification of the learning and validation set compounds as low, medium and high risk should be done prior to model development, therefore the performance of a more granular classification requires additional study. In the interim, the cardiac risk algorithm classification of a compound as TdP(+)/(−) should be interpreted along with its associated probability.

Finally, the predictive performance of any classification model depends not only on the algorithm utilized for the model development but also the data quality. Discussion around the role of both elements is out of scope of the current manuscript, yet it can be hypothesized that data quality is at least equally, if not more, important than the algorithm used. As was recently discussed, TdP risk classifications vary between sources and there is no conclusive information on TdP risk for multiple known drugs [43]. It is likely that alternative classifications for certain drugs, such as those whose classification has been modified by CredibleMeds^®^ could have changed the modeling results, thereby highlighting an inherent risk of relying on a static “snapshot” of a dynamic classification system that is continuously updated with gained clinical experience.

The main limitations of the present study are listed below:

- A single chemical entity was utilized for the model development; to analyze and predict the influence of multiple chemicals (i.e., drugs and/or their metabolites), and mechanistic models allowing for the interaction
- In vitro data quality—heterogeneous sources were used at the model development stage, all of them from the publicly available literature
- Lack of non-drug parameters influencing drugs torsadogenicity (i.e., interacting drugs, diseases, physiological parameters and their variability).

In conclusion, an in silico modelling and simulation approach that considered ECG changes beyond QT was proposed as a tool for the cardiac safety assessment. The cardiac safety simulator connected with the Simcyp platform was used as the simulation platform. Despite the potential sources of uncertainty, the combined PBPK-PD model was able to reasonably extrapolate in vitro data to the in vivo situation to predict clinical cardiac consequences of drugs, including anti-TB agents. Such an approach allows the testing of clinical scenarios early in drug development programs, thus facilitating earlier go/no-go decisions. This is of specific importance for the evaluation of novel anti-TB drugs in development, given the potential association of anti-TB agents with QT prolongation and the need for combination therapies for effective treatment. Therefore, future work will expand this methodology to support the selection of combination regimen components based on overall TdP probability.

## Electronic supplementary material

Below is the link to the electronic supplementary material.

Supplementary material 1 (XLSX 67 kb)

## Abbreviations

ADTree: Alternating decision tree
APD: Action potential duration
BDMM: Biophysically-detailed myocyte models
CAD: Cationic amphiphilic drug
CDISC: Clinical trial data interchange standards consortium
CPTR: Critical path to TB drug regimen initiative
CSS: Cardiac safety simulator
DDI: Drug-drug interaction
ECG: Electrocardiogram
EMW: Electromechanical window
hERG: Human ether-a-go–go-related gene
ORD: O’Hara-Rudy models
PBPK: Physiologically based pharmacokinetics
PD: Pharmacodynamics
PK: Pharmacokinetics
QTc: Heart rate corrected QT interval
ROC: Receiver-operator curve
RR: Time gap between the peaks of the QRS complex of the ECG wave
TB: Tuberculosis
TdP: Torsade de pointes
WEKA: Waikato environment for knowledge analysis

## References

[1] World Health Organization (2015) Global tuberculosis report. WHO, Geneva. http://www.who.int/tb/publications/global_report/gtbr15_main_text.pdf. Accessed 19 Apr 2016

[2] Hanna D, Romero K, Schito M (2017). Advancing TB drug regimen development through innovative quantitative translational pharmacology methods and approaches. Int J Infect Dis.

[3] Pugsley MK, Authier S, Curtis MJ (2008). Principles of safety pharmacology. Br J Pharmacol.

[4] Waring MJ (2015). An analysis of the attrition of drug candidates from four major pharmaceutical companies. Nat Rev Drug Discov.

[5] Ferria N, Siegl P, Corsini A, Herrmann J, Lerman A, Benghozi R (2013). Drug attrition during pre-clinical and clinical development: understanding and managing drug-induced cardiotoxicity. Pharmacol Ther.

[6] Redfern WS (2010). Impact and frequency of different toxicities throughout the pharmaceutical life cycle. Toxicologist.

[7] Verma S, Kaplowitz N, Kaplowitz N, DeLeve LD (2013). Hepatotoxicity of antituberculosis drugs. Drug-induced liver disease.

[8] Kowey PR, Malik M (2007). The QT interval as it relates to the safety of non-cardiac drugs. Eur Heart J.

[9] Stockbridge N, Morganroth J, Shah RR, Garnett C (2013). Dealing with global safety issues: was the response to QT-liability of non-cardiac drugs well coordinated?. Drug Saf.

[10] Gintant G, Sager PT, Stockbridge N (2016). Evolution of strategies to improve preclinical cardiac safety testing. Nat Rev Drug Discov.

[11] Cummins Lancaster M, Sobie EA (2016). Improved prediction of drug-induced Torsades de pointes through simulations of dynamics and machine learning algorithms. Clin Pharmacol Ther.

[12] Yap CW (2004). Prediction of torsade-causing potential of drugs by support vector machine approach. Toxicol Sci.

[13] He Y (2012). Determination of torsade-causing potential of drug candidates using one-class classification and ensemble modelling approaches. Curr Drug Saf.

[14] Johannesen L (2016). Differentiating drug-induced multichannel block on the electrocardiogram: randomized study of dofetilide, quinidine, ranolazine, and verapamil. Clin Pharmacol Ther.

[15] Wisniowska B, Polak S (2016). Virtual clinical trial towards polytherapy safety assessment—combination of PBPK/PD based modelling and simulation approach with DDIs involving terfenadine as an example. J Pharm Sci.

[16] Colatsky T (2016). The comprehensive in vitro proarrhythmia assay (CiPA) initiative—update on progress. J Pharmacol Toxicol Methods.

[17] ten Tusscher KH, Noble D, Noble PJ, Panfilov AV (2004). A model for human ventricular tissue. Am J Physiol Heart Circ Physiol.

[18] ten Tusscher KH, Panfilov AV (2006). Alternans and spiral breakup in a human ventricular tissue model. Am J Physiol Heart Circ Physiol.

[19] O’Hara T, Virág L, Varró A, Rudy Y (2011). Simulation of the undiseased human cardiac ventricular action potential: model formulation and experimental validation. PLoS Comput Biol.

[20] Tox-portal. www.tox-portal.net. Accessed 08 June 2017

[21] Abbasi M, Patel N, Small B, Jamei M, Polak S (2016). Early assessment of pro-arrhythmic risk of drugs using the in vitro data and single cell based in silico models: proof of concept. Toxicol Mech Methods.

[22] Glinka A, Polak S (2015). QTc modification after risperidone administration—insight into the mechanism of action with use of the modeling and simulation at the population level approach. Toxicol Mech Methods.

[23] Lu HR, Yan GX, Gallacher DJ (2013). A new biomarker–index of cardiac electrophysiological balance (iCEB)–plays an important role in drug-induced cardiac arrhythmias: beyond QT-prolongation and Torsades de pointes (TdPs). J Pharmacol Toxicol Methods.

[24] Witten IH, Eibe F, Hall MA (2011). Data mining: practical machine learning tools and techniques.

[25] Polak S, Fijorek K, Glinka A, Wisniowska B, Mendyk A (2012). Virtual population generator for human cardiomyocytes parameters. In silico drug cardiotoxicity assessment. Toxicol Mech Methods.

[26] Polak S, Fijorek K (2012). Inter-individual variability in the pre-clinical drug cardiotoxic safety assessment analysis of the age—cardiomyocytes electric capacitance dependence. J Cardiovasc Transl Res.

[27] Fijorek K, Patel N, Klima Ł, Stolarz-Skrzypek K, Kawecka-Jaszcz K, Polak S (2013). Age and gender dependent heart rate circadian model development and performance verification on the proarrhythmic drug case study. Theor Biol Med Model.

[28] Fijorek K, Püsküllüoglu M, Polak S (2013) Circadian models of serum potassium, sodium and calcium concentrations in healthy individuals, and their application to cardiac electrophysiology simulations at individual level. Computational and Mathematical Methods in Medicine Article ID 429037, 8 pages 10.1155/2013/429037 PMC3775438 24078832

[29] Fijorek K (2014). Model of the distribution of diastolic left ventricular posterior wall thickness in healthy adults and its impact on the behavior of a string of virtual cardiomyocytes. J Cardiovasc Transl Res.

[30] Robyns T (2016). Evaluation of index of cardio-electrophysiological balance (iCEB) as a new biomarker for the identification of patients at increased arrhythmic risk. Ann Noninvasive Electrocardiol.

[31] Guns PJ, Johnson DM, Weltens E, Lissens J (2012). Negative electro-mechanical windows are required for drug-induced Torsades de pointes in the anesthetized guinea pig. J Pharmacol Toxicol Methods.

[32] CredibleMeds https://crediblemeds.org/. Accessed 07 June 2017

[33] Woosley RL, Black K, Heise CW, Romero K (2018). CredibleMeds.org: what does it offer?. Trends Cardiovasc Med.

[34] Freund Y, Mason L (1999). The alternating decision tree learning algorithm. Proceeding of the sixteenth international conference on machine learning.

[35] Merle CS (2014). OFLOTUB/gatifloxacin for tuberculosis project a four-month gatifloxacin-containing regimen for treating tuberculosis. N Engl J Med.

[36] Haddad PM, Anderson IM (2002). Antipsychotic-related QTc prolongation, torsade de pointes and sudden death. Drugs.

[37] Detrol—tolterodine tartrate tablets https://www.accessdata.fda.gov/drugsatfda_docs/label/2009/020771s022lbl.pdf. Accessed 07 June 2017

[38] Antzelevitch C (2004). Electrophysiologic properties and antiarrhythmic actions of a novel antianginal agent. J Cardiovasc Pharmacol Ther.

[39] Chaitman BR (2006). Ranolazine for the treatment of chronic angina and potential use in other cardiovascular conditions. Circulation.

[40] Haverkamp W, Kruesmann F, Fritsch A, van Veenhuyzen D, Arvis P (2012). Update on the cardiac safety of moxifloxacin. Curr Drug Saf.

[41] Tulkens PM, Kruesmann F (2012). Moxifloxacin safety: an analysis of 14 years of clinical data. Drugs R D.

[42] Nguyen TV, Cao TB, Akkerman OW, Tiberi S, Vu DH, Alffenaar JW (2016). Bedaquiline as part of combination therapy in adults with pulmonary multi-drug resistant tuberculosis. Expert Rev Clin Pharmacol.

[43] Wisniowska B, Polak S (2017). So am I or am I not proarrhythmic? Comparison of various classifications of drugs TdP propensity. Drug Discov Today.

